# Newly Designed Break-Apart and *ASPL*-*TFE3* Dual-Fusion FISH Assay Are Useful in Diagnosing Xp11.2 Translocation Renal Cell Carcinoma and *ASPL*-*TFE3* Renal Cell Carcinoma: A STARD-Compliant Article

**DOI:** 10.1097/MD.0000000000000873

**Published:** 2015-05-21

**Authors:** Xiancheng Chen, Yang Yang, Weidong Gan, Linfeng Xu, Qing Ye, Hongqian Guo

**Affiliations:** From the Departments of Urology (XC, YY, WG, LX, HG) and Pathology (QY), Nanjing Drum Tower Hospital, the Affiliated Hospital of Nanjing University Medical School, Nanjing, China

## Abstract

The diagnosis of Xp11.2 translocation renal cell carcinoma (tRCC), which relies on morphology and immunohistochemistry (IHC), is often either missed in the diagnosis or misdiagnosed. To improve the accuracy of diagnosis of Xp11.2 tRCC and *ASPL*-*TFE3* renal cell carcinoma (RCC), we investigated newly designed fluorescence in situ hybridization (FISH) probes (diagnostic accuracy study).

Based on the genetic characteristics of Xp11.2 tRCC and the *ASPL*-*TFE3* RCC, a new break-apart *TFE3* FISH probe and an *ASPL*-*TFE3* dual-fusion FISH probe were designed and applied to 65 patients with RCC who were <45 years old or showed suspicious microscopic features of Xp11.2 tRCC in our hospital. To test the accuracy of the probes, we further performed reverse transcriptase–polymerase chain reaction (PCR) on 8 cases for which frozen tissues were available.

Among the 65 cases diagnosed with RCC, TFE3 IHC was positive in 24 cases. Twenty-two cases were confirmed as Xp11.2 tRCC by break-apart TFE3 FISH, and 6 of these cases were further diagnosed as *ASPL*-*TFE3* RCC by *ASPL*-*TFE3* dual-fusion FISH detection. Importantly, reverse transcriptase–PCR showed concordant results with the results of FISH assay in the 8 available frozen cases.

The break-apart and *ASPL*-*TFE3* dual-fusion FISH assay can accurately detect the translocation of the *TFE3* gene and *ASPL*-*TFE3* fusion gene and can thus serve as a valid complementary method for diagnosing Xp11.2 tRCC and *ASPL*-*TFE3* RCC.

## INTRODUCTION

Xp11.2 translocation renal cell carcinoma (tRCC) was classified as a new type of renal cell carcinoma (RCC) in the 2004 World Health Organization classification system and is characterized by various fusions of the transcription factor E3 (*TFE3*) gene.^1,2^ Five fusion gene patterns have been identified so far: t(X;17)(p11.2;q25)generates the *ASPL*-*TFE3* fusion gene^3^; t(X;1)(p11.2;q21) generates the *PRCC*-*TFE3* fusion gene^4^; t(X;17)(p11.2;q23) generates the *CLTC*-*TFE3* fusion gene^5^; t(X;1)(p11.2;p34) generates the *PSF*-*TFE3* fusion gene^6^; and inv(X)(p11;q12) generates the *NonO*-*TFE3* fusion gene.^6^*ASPL*-*TFE3* and *PRCC*-*TFE3* RCC are the most common types of Xp11.2 tRCC. Furthermore, *ASPL*-*TFE3* RCC is more malignant than the other subtypes are.^7^ The diagnosis of *ASPL*-*TFE3* RCC is always accompanied by lymphatic and distant organ metastasis, and the overall survival is poor.^8^

Currently, the diagnosis of Xp11.2 tRCC in hospitals relies primarily on pathologic morphology and immunohistochemistry (IHC) evidence. Xp11.2 tRCC typically has a clear or acidophilic voluminous cytoplasm, hyaline stroma, and psammoma bodies. Furthermore, the growth of Xp11.2 tRCC usually follows an alveolar, nest, or papillary pattern.^9^ The morphological identification of Xp11.2 tRCC has an overlap with clear cell RCC and papillary RCC; thus, IHC evidence is necessary to identify Xp11.2 tRCC. TFE3 IHC was believed to have a high specificity and sensitivity for diagnosis,^10^ but its predictive value was found to be only 12% in a recent study by Klatte et al.^11^ Reverse transcription–polymerase chain reaction (PCR) and cytogenetic karyotypic analysis are tools that can be used to ascertain specific types of genetic changes in tumor cells.^12^ However, fresh tumor tissues are not always available, and reverse transcriptase–PCR occasionally fails to detect the translocation due to the instability and rapid degradation of RNA in a formalin-fixed, paraffin-embedded (FFPE) block.

Fluorescence in situ hybridization (FISH) assay has a notable advantage of detecting the translocation and duplication of genes, particularly for interphase cells and complicated karyotypes. Herein, we report a new break-apart FISH probe for diagnosing Xp11.2 tRCC and a novel *ASPL*-*TFE3* dual-fusion FISH probe for diagnosing *ASPL*-*TFE3* RCC.

## MATERIALS AND METHODS

### Case Selection

A total of 983 RCC patients were reviewed at Nanjing Drum Tower Hospital from January 2007 to October 2014, and the review included their medical records and outcomes. Among these patients, there were 65 RCC cases who were <45 years old or showed suspicious microscopic features of Xp11.2 tRCC. Twenty typical cases of clear RCCs and 20 cases of papillary RCCs were also collected as control groups. All of the FFPE tissues of those cases were reviewed and prepared for IHC and FISH assay. In addition, reverse transcriptase–PCR tests were performed on 8 of these 65 cases for which frozen tissues were available. This study was approved by the institutional review board of Nanjing Drum Tower Hospital.

### TFE3 IHC

Four-micrometer-thick tumor tissue sections were prepared for TFE3 IHC in all 65 suspected Xp11.2 tRCC cases. After deparaffinization, the FFPE sections were treated with 0.3%H_2_O_2_ for 10 minutes at room temperature to block endogenous peroxidase activity. The rabbit anti-TFE3 monoclonal antibody (prediluted, ZSGB-BIO, Beijing, China) was used as the primary antibody, and the sample sections were incubated at 4°C overnight. After a washing step, the sample sections were incubated with biotinylated anti-rabbit IgG (ZSGB-BIO) for 20 minutes at room temperature.^11^

### Reverse Transcription–PCR

Reverse transcription–PCR was performed on the 8 available frozen tissues to detect the existence of fusion genes by using five pairs of targeted RNA primers, as reported previously.^12^ Total RNA was extracted via TRIzol standard procedures, and RNA was converted to complementary cDNA by random primers (TaKara Biotechnology, Dalian, china) and EasyScript Reverse Transcriptase (TransGen Biotech, Beijing, China). The *ASPL* forward primer was applied to detect the *ASPL*-*TFE3* fusion gene: 5′-AAAGAAGTCCAAGTCGGGCCA-3′ with *TFE3* reverse primer: 5′-CGTTTGATGTTGGGCAGCTCA-3′. The *PRCC* forward primer was applied to detect the *PRCC*-*TFE3* fusion gene: 5′-CCAAGCCAAAGAAGAGGA-3′ with *TFE3* reverse primer: 5′-AGTGTGGTGGACAGGTACTG-3′. The *PSF* forward primer was applied to detect the *PSF*-*TFE3* fusion gene: 5′-TGGTGGTGGCATAGGTTATG-3′ with *TFE3* reverse primer: 5′-CGTTTGATGTTGGGCAGCTC-3′. The *NonO* forward primer was applied to detect the *NonO*-*TFE3* fusion gene: 5′-GAGAAACTAGACACAGCAAC-3′ with *TFE3* reverse primer: 5′-CTTTCTTCTGCCGTTCCTTC-3′. The *CLTC* forward primer was applied to detect the *CLTC*-*TFE3* fusion gene: 5′-AGTCGCGTTGTTGGAAAGTATTGTG-3′ with TFE3 reverse primer 5′-CAAAAGGGCCTTTGCCTCGGTC-3′. PCR amplification was performed in a volume of 20 μL under the following conditions: 95°C for 10 minutes; followed by 40 cycles of 95°C for 45 seconds, 60°C for 45 seconds, and 72°C for 60 seconds; and a final 10-minute extension at 72°C.

PCR products were separated in 1.5% agarose gels and extracted using the QIA quick Gel Extraction Kit (Qiagen Inc, Hilden, Germany), and sequence analyses were completed on a 3130 Genetic Analyzer (Applied Biosystems, Foster City, CA). The resulting PCR product sequences were compared by using BLAST (http://blast.ncbi.nlm.nih.gov/Blast.cgi).

### DNA Probe Design

**FIGURE 1:**
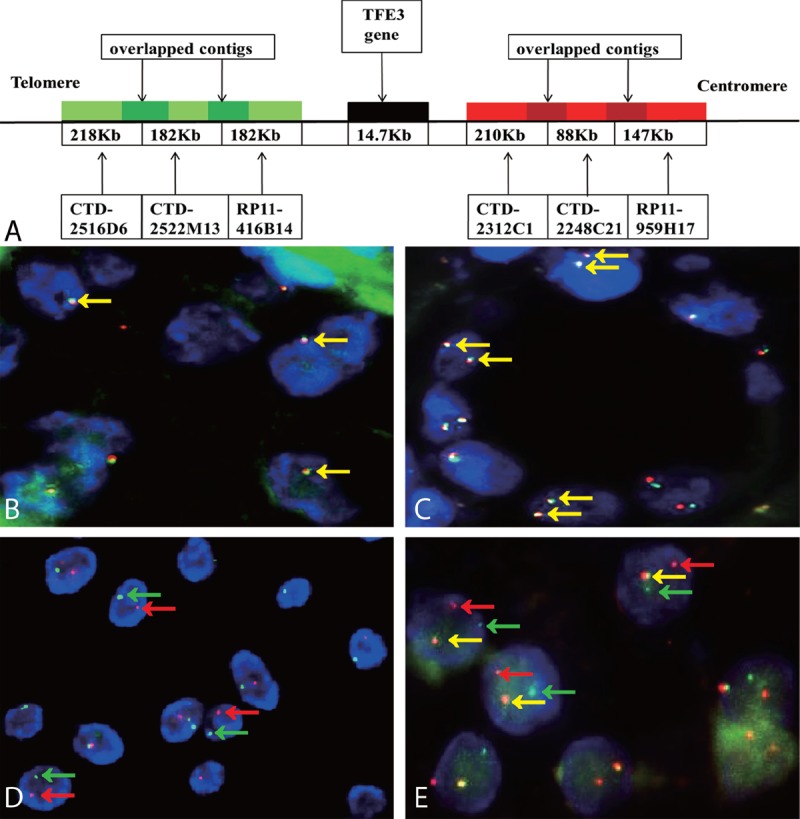
(A) schematic representation of the *TFE3* break-apart probe. Bacterial artificial chromosomes (BACs) labeled with a green fluorescence signal were located upstream of the *TFE3* gene, and BACs with red labels were downstream. B and C show typical negative results of *TFE3* break-apart FISH assay in males (1F, yellow arrowheads) and females (2F, yellow arrowheads)(×1000). D shows the typical positive result in males (1G1R, red and green arrowheads), indicating the translocation of the only 1 X chromosome (×1000); E shows a typical positive result in Xp11.2 translocation renal cell carcinoma tissues in females (1G1R1F, yellow, red, and green arrowheads), indicating the translocation of 1 X chromosome and another normal X chromosome (×1000).

**FIGURE 2:**
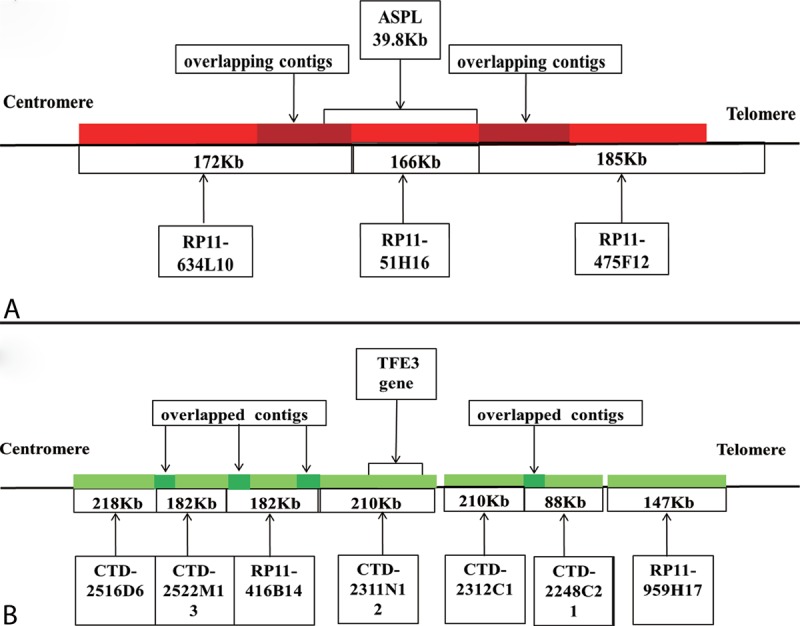
A schematic representation of the *ASPL*-*TFE3* dual-fusion probe. (A) BACs labeled with red fluorescence covered the entire ASPL gene. (B) Bacterial artificial chromosomes (BACs) labeled with green fluorescence covered the entire TFE3 gene.

1. Break-apart probe design: Suitable bacterial artificial chromosomes (BACs) for the break-apart probe were selected at http://genome.ucsc.edu/ following the main requirements that the BACs located on the same side of the *TFE3* gene should have close or overlapping contig with adjacent BAC and the BACs between the sides of the *TFE3* gene should be completely separated. Six BACs located on the 2 sides surrounding the *TFE3* gene were chosen: CTD-2516D6, CTD-2522M13, and RP11-416B14 were located on the telomeric side of the *TFE3* gene, and CTD-2312C1, CTD-2248C21, and RP11-959H17 were located on the centromeric side of the *TFE3* gene. DNA was extracted from the BAC (Invitrogen, Carlsbad, CA, Carlsbad, USA) after being cultured using a Qiagen Plasmid Maxi Kit (Qiagen). The BACs that were located on the telomeric *TFE3* gene were labeled with green fluorescein with fluorescein-12-dUTP (Roche Diagnostics GmbH, Mannheim, Germany), and the centromeric BACs were labeled with red fluorescein with tetramethylrhodamine-5-dUTP (Roche) via nick translation (Figure 1).

2. Dual-fusion dual-color probe design: After being selected from http://genome.ucsc.edu/, RP11-634L10, RP11-51H16, and RP11-475F12 were labeled with tetramethylrhodamine-5-dUTP as red fluorescein, were located on the long arm of chromosome 17 and covered the entire *ASPL* gene. CTD-2311N12, RP11-416B14, CTD-2522M13, CTD-2516D6, CTD-2312C1, CTD-2248C21, and RP11-959H17 were located on the short arm of the X chromosome and covered the entire *TFE3* gene. They were labeled with fluorescein-12-dUTP as green fluorescein (Figure 2).

Both probes were hybridized with normal blood peripheral lymphocyte metaphase cells to observe their actual location and integrity. The FISH probe was mixed with purified DNA with human Cot-1 DNA, hybridization buffer, and purified water and was stored at −20°C in the dark.

### FISH Assay

1. FISH experimental procedure: Three-μm-thick sections from FFPE tumor tissue blocks were deparaffinized with xylene for 30 minutes and washed with absolute ethanol for 10 minutes. Sections were rehydrated in 100%, 85%, and 70% ethanol in turn for 3 minutes and were then air dried. Sections were digested with 10 μL pepsin (4 mg/mL, 0.02 M HCl; Sigma-Aldrich, Beijing, China) at 37°C for 3 to 5 minutes and washed with 2× saline–sodium citrate (SSC) for 5 minutes. Subsequently, they were dehydrated in 70%, 85%, and 100% ethanol in turn for 3 minutes and were then air dried. Hybridization was performed with the new FISH probe, denatured at 85°C for 5 minutes, and incubated overnight at 37°C. The sections were immersed in 2× SSC for 10 minutes and in 0.1% NP-40/2× SSC for 5 minutes at 37°C. Then, the sections were immersed in 70% ethanol for 3 minutes and air dried. The nuclei were counterstained with 5 μL 4,6-diamidino-2-phenylindole (DAPI). After hybridization, all slides were kept away from light.

2. FISH result evaluation was conducted using a Olympus BX51TRF fluorescence microscope (Olympus, Tokyo, Japan) with 3 filters (DAPI/FITC/TexasRed) and the FISH analysis software (Imstar, Paris, France). Two experienced pathologists (Jun Yang and Xiaohong Pu) assessed the results of the FISH and were blinded to the results of the other tests. The FISH assay is effective only when clear FISH signals are observed in >100 nonoverlapping nuclei.

For the break-apart probe, 2 typical signal patterns could be detected in cell interpretation. For the fusion signals, yellow or adjoined green–red signal patterns were considered normal signals and indicated that *TFE3* gene was not rearranged. For the break-apart signals, the *TFE3* gene was considered to be rearranged when the green and red signals were separated by a distance >1 signal diameter, which is 1 characteristic of Xp11.2 tRCC. The number of fusion signals relies on the quantity of X chromosomes: females have 2 fusion signals (2F), whereas males have only 1 (1F). Females with Xp11.2 tRCC have 1 fusion signal and a pair of split green–red signals (1R1R1F), which represents 1 normal X chromosome and 1 rearranged X chromosome. Males with Xp11.2 tRCC have only 1 pair of split green–red signals due to the single X chromosome (1G1R) (Figure 1). Only the split green–red signals greater than 10% that emerged in the observed tumor nuclei were considered to be a positive result in the TFE3 break-apart FISH assay.

**FIGURE 3:**
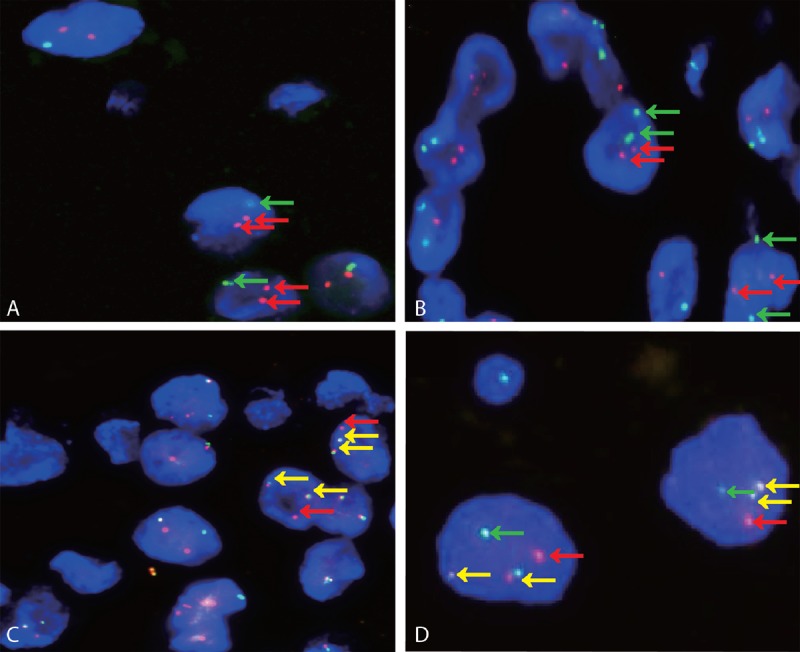
Both A and B show typical negative results of *ASPL*-*TFE3* dual-fusion FISH assay in males (1G2R, red and green arrowheads) and females (2G2R, red and green arrowheads) (×1000). C and D show representative positive results in males (2F1R, yellow and green arrowheads) and females (2F1G1R, yellow, red, and green arrowheads) (×1000).

For the dual-fusion probe, 2 typical signal patterns could be detected in cell interpretation. Fusion signal (yellow and abutting green-red signals) emerged when a new *ASPL*-*TFE3* fusion gene was formed. We marked the entire *ASPL* gene as a red signal and the entire *TFE3* gene as a green signal. When the 2 fusion signals appeared simultaneously, 2 *ASPL*-*TFE3* fusion genes were identified; thus, the reciprocal translocation between the *ASPL* and *TFE3* genes could be confirmed. In contrast, 1 fusion signal represented an unbalanced translocation. When split green–red signals appeared, the green and red signals represented the *TFE3* gene in chromosome X and the *ASPL* gene in chromosome 17. Thus, females have 2 split green signals and 2 red signals in 1 nucleus, whereas males have only 1 green signal and 2 red signals. The threshold value for dual-fusion probe was set at 2% (Figure 3).

## RESULTS

### Patients

According to the 2004 WHO classification diagnostic criteria, 22 cases were diagnosed as Xp11.2 tRCC, as diagnosed by pathologic morphology, TFE3 IHC, and *TFE3* break-apart FISH assay. Based solely on the pathologic morphology, those cases could be divided into clear cell RCC (8 cases), papillary RCC (7 cases), unclassified RCC (1 case), and Xp11.2RCC (6 cases). The patients were identified as 13 females and 9 males (ratio of female to male patients was 1.4:1), with a median age of 27 years (range 3–51 y). Compared with the initial diagnosis of those 65 cases dependent on the pathologic morphology and IHC, 2 cases were misdiagnosis and 6 cases of Xp11.2 tRCC were missed diagnosis. All of the positive results in this study, including initial diagnosis, TFE3 IHC, *TFE3* break-apart FISH, *TFE3* dual-fusion FISH, final diagnosis, and outcome are summarized in Table 1.

**TABLE 1:**
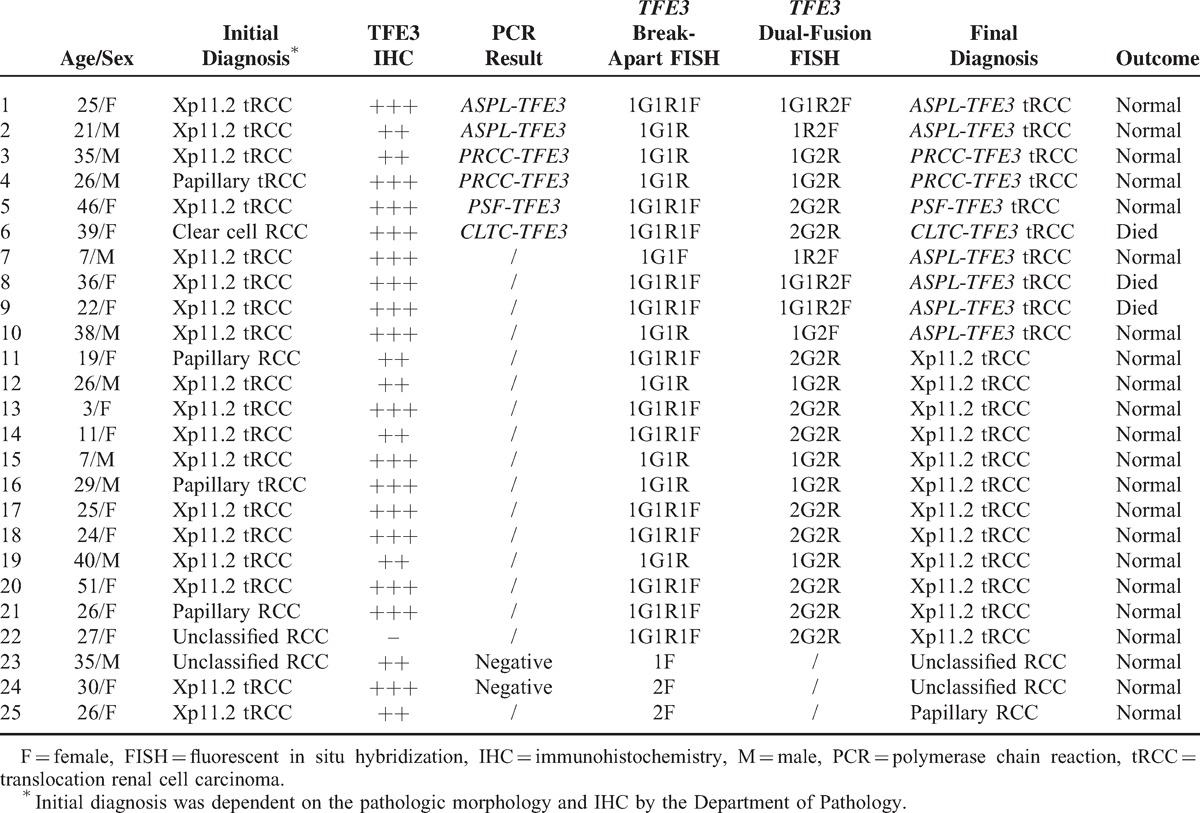
Summary of the Results of Initial Diagnosis, TFE3 IHC, *TFE3* Break-Apart FISH, *ASPL*-*TFE3* Dual-Fusion FISH and Outcome

### TFE3 IHC, Break-Apart Probe and Dual-Fusion Probe

TFE3 IHC was performed on the 65 patients who were younger than 45 years old or showed suspicious microscopic features of Xp11.2 tRCCs, and 24 of these cases were found to be positive. Break-apart FISH assay was performed on those 65 cases and on the 20 clear RCCs and the 20 papillary RCCs, which served as control groups. There were 22 cases that positively predicted the translocation of *TFE3* gene among the 65 cases, whereas the other 43/65 cases and the control groups were negative. However, among the 22 cases of Xp11.2 tRCCs, 21/22 cases were positive for TFE3 IHC. *ASPL*-*TFE3* dual-fusion FISH assay was performed on those 22 cases of Xp11.2 tRCCs and on the 20 cases of papillary RCCs and clear cell RCCs. *ASPL*-*TFE3* RCC was diagnosed in 6 cases, which showed dual-fusion signals in the *ASPL*-*TFE3* dual-fusion FISH assay, and the other 16/22 cases and control groups were negative.

### Validation of Break-Apart Probe and *ASPL*-*TFE3* Dual-Fusion Probe

**FIGURE 4:**
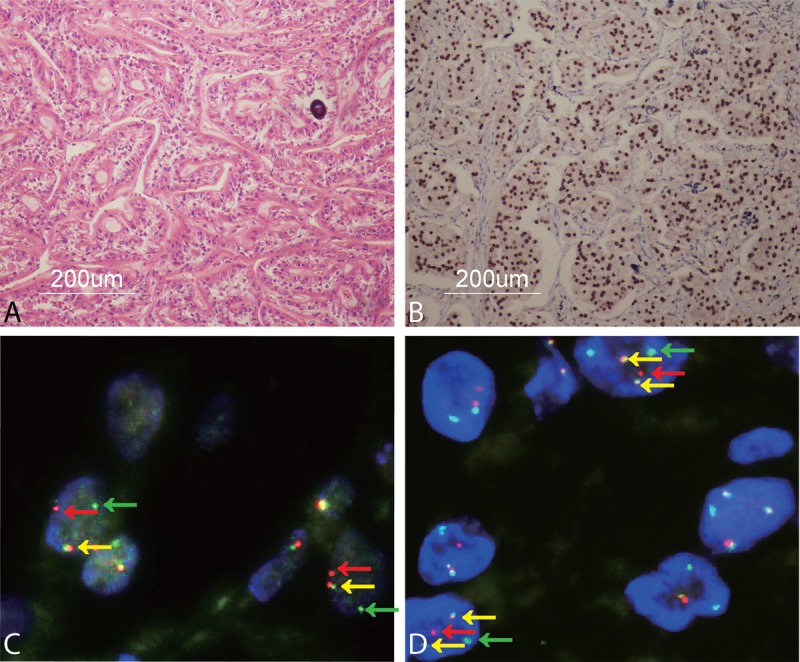
A shows typical histopathology of *ASPL*-*TFE3* RCC including voluminous cytoplasm, growth in a papillary pattern, and a psammoma body in case 1 (×100); B shows strong TFE3 IHC nuclear staining (×100); C shows a *TFE3* break-apart FISH assay characterized by separated red and green signals (red and green arrowheads) and a fusion signal (yellow arrowheads) in tumor cell nuclei in case 1 (×1000); D shows ASPL-TFE3 dual-fusion FISH assay displaying 2 fusion signals (yellow arrowheads) in each tumor cell nucleus and 2 separated red and green signals (red and green arrowheads) in case 1 (×1000).

**FIGURE 5:**
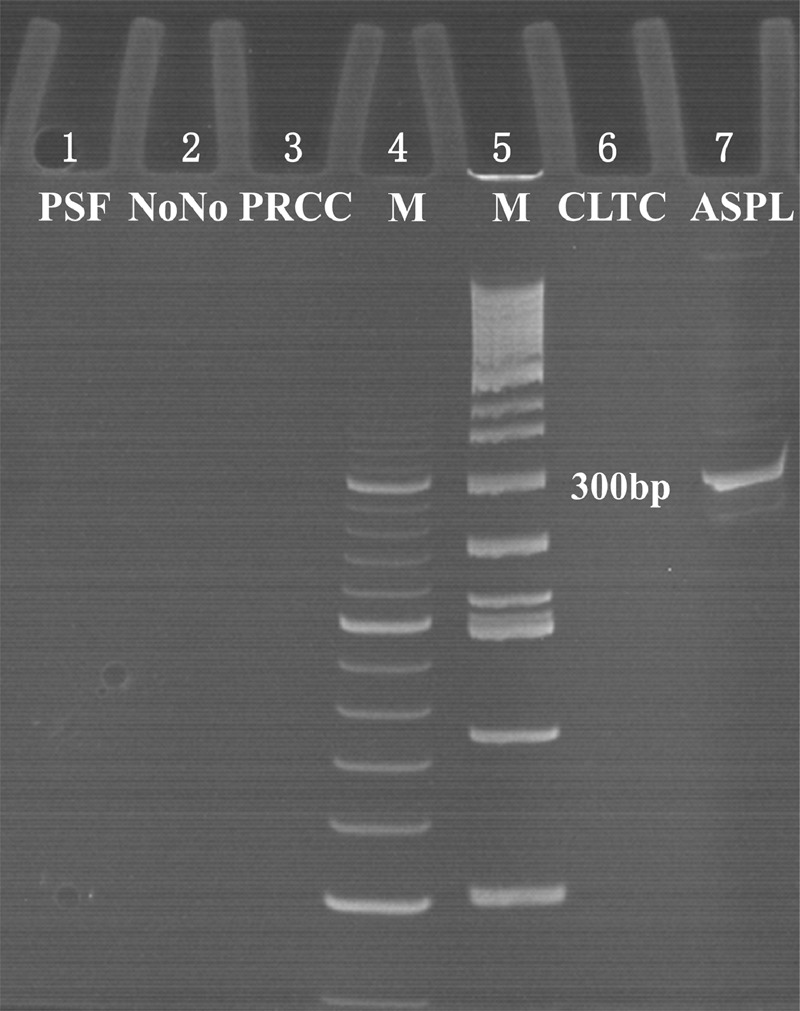
The results of reverse transcriptase–PCR showed a 310 bp targeted *ASPL*-*TFE3* transcript in lane 7 in case 1; lanes 4 and 5 were markers; and lanes 1, 2, 3, and 6 were negative reverse transcriptase–PCR results of *PSF*-*TFE3*, *NonO*-*TFE3*, *PRCC*-*TFE3* and *CLTC*-*TFE3*, respectively.

**FIGURE 6:**
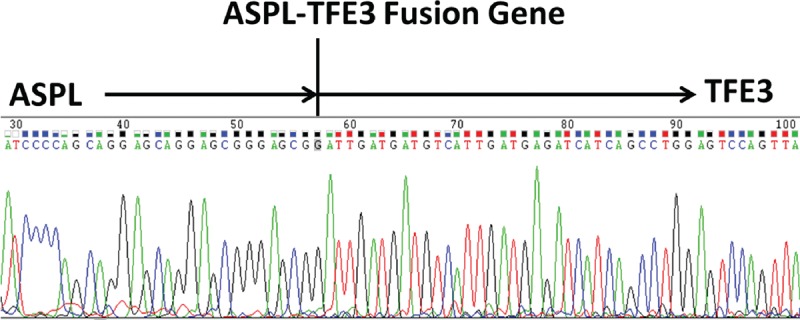
Sequencing of reverse transcriptase–PCR results showed the *ASPL*-*TFE3* fusion gene in case 1.

Reverse transcription–PCR was performed on the 8 cases for which frozen tissues were available and confirmed 6 cases of Xp11.2 tRCCs. Moreover, 2 of those 6 cases were *ASPL*-*TFE3* RCCs. Compared with the results of the FISH assay, the 6 cases of Xp11.2 tRCCs that were confirmed by PCR were positive in the break-apart FISH assay and the 2 cases of *ASPL*-*TFE3* RCC confirmed by PCR showed concordant results with the *ASPL*-*TFE3* dual-fusion FISH assay. (Figures 4–6).

## DISCUSSION

Xp11.2 tRCC is being increasingly recognized and has attracted broad attention in recent years. Xp11.2 tRCC was classified as “the microphthalmia transcription factor/transcription factor E (MiTF/TFE) family translocation carcinoma” by Argani and Ladanyi.^13^ Other MiTF/TFE family translocation carcinoma members include alveolar soft part sarcoma (ASPS) and perivascular epithelioid cell tumors (PEComas). Xp11.2 tRCC mainly occurs in juveniles and young adults and has an incidence of 30% in pediatrics, 15% in young adults (younger than 45 years), and 1.5% in adults.^14–16^ There is not yet a widely accepted standard therapy, and conventional chemotherapy and radiotherapy may be invalid in this carcinoma. Radical nephrectomy is recommended if surgery is feasible,^17^ and targeted therapies such as vascular endothelial growth factor receptor-targeted and Met inhibitor therapy have been shown to be only partly effective.^18–20^ The clinical characterization, diagnosis, auxiliary treatment, and prognosis of Xp11.2 tRCC differ from those used in other RCC types, which is why Xp11.2 tRCC requires a more accurate diagnosis and identification compared with other subtypes of RCC.

As indicated from our study, simple pathologic morphology and TFE3 IHC results may result in either missed diagnosis or misdiagnosis in cases of Xp11.2 tRCC. In this study, only 6 cases showed distinctive morphology of Xp11.2 tRCC, whereas most cases overlapped with clear cell RCC and papillary RCC. The TFE3 IHC result was generally consistent with the FISH results. However, the false-positive (7.0%) and false-negative rates (4.5%) must not be ignored. Hence, further tests are necessary to validate the TFE3 IHC results to obtain more accurate diagnosis. The *TFE3* break-apart FISH assay has been proven to be an effective tool for the diagnosis of Xp11.2 RCC.^7,21–25^ Compared with previous probes, we applied more BACs to promote the signal strength and maintained the same side for adjoined BACs to prevent signal scattering. The results of FISH assay that was conducted with the break-apart and dual-fusion probes are in agreement with the PCR results, which confirm the reliability of our probes.

In this study, we observed that 2/3 patients who died from this disease due to multiple metastasis have *ASPL*-*TFE3* RCC. According to Komai et al's study,^26^ 2 cases with *ASPL*-*TFE3* RCC displayed visceral metastases, and 1 died of the disease. In Malouf et al's study,^8^ 2 patients with *ASPL*-*TFE3* fusion had more aggressive progression compared with those who had other fusion genes. Furthermore, Ellis et al reviewed all of the published *ASPL*-*TFE3* as well as *PRCC*-*TFE3* RCC cases and found that *ASPL*-*TFE3* RCC usually presents at an advanced stage.^27^ Ellis's group found that regional lymph nodes were involved in 24 of the 32 *ASPL*-*TFE3* RCC cases and that patients who had distant metastasis also displayed *ASPL*-*TFE3* fusion genes. The rate was distinctly higher than that of *PRCC*-*TFE3* RCCs. It has been shown that the presence of lymph nodes and advanced stage at diagnosis contribute to the poor prognosis in *ASPL*-*TFE3* RCC cases.^8^ The disease development process also differs between children and adults. It is an indolent process in children, but adult patients normally experience a more aggressive process.^28^ For Xp11.2 tRCC, it is necessary to distinguish the *ASPL*-*TFE3* RCC from other types.

The novel *ASPL*-*TFE3* dual-fusion probe is designed here for the first time using in diagnosing Xp11.2 tRCC. According to the design strategy, BACs cover the entirety of the *ASPL* and *TFE3* genes. Two fusion signals emerge simultaneously in 1 nucleus, indicating that *ASPL*-*TFE3* fusion genes have been formed by balanced translocation. The fusion signals would emerge in tissues with nonreciprocal translocations of the *ASPL* and *TFE3* genes. Unbalanced translocation of *ASPL* and *TFE3* genes had been identified in Xp11.2 tRCC before,^7^ and only a dual-fusion probe can differentiate the nonreciprocal translocation from the reciprocal translocation of the *ASPL* and *TFE3* genes. This novel dual-fusion probe can accurately distinguish the balanced and unbalanced translocation of the *TFE3* gene, which is the superior to single fusion probe strategy.^7^

Furthermore, our new *TFE3* break-apart and *ASPL*-*TFE3* dual-fusion probes can be applied diagnostically in other tumors that harbor translocation of the *TFE3* gene and the *ASPL*-*TFE3* fusion gene. PEComas have been found to have *TFE3* gene rearrangement and to harbor a *PSF*-*TFE3* gene fusion^29^; thus, the *TFE3* break-apart probe could be applied to detect the translocation of the *TFE3* gene. ASPS could harbor an unbalanced translocation of the *TFE3* gene, which results from der(17)t(X; 17)(p11.2; q25).^30,31^ Therefore, a split green signal pattern would occur when ASPS is detected by the break-apart probe FISH assay, whereas a solitary fusion signal pattern could be detected via the dual-fusion probe FISH assay. By combining the *TFE3* break-apart and *ASPL*-*TFE3* dual-fusion probes, the genetic changes in ASPS can be clearly confirmed.

In summary, we have designed a novel *ASPL*-*TFE3* dual-fusion probe and a new *TFE3* break-apart probe and put them into practice. Compared with the PCR results, we confirmed that both new probes are accurate for detecting Xp11.2 tRCC and *ASPL*-*TFE3* RCC. Furthermore, these FISH assays can be used as an adjunctive method to improve the accuracy of diagnosis for Xp11.2 tRCC, ASPS, PEComas, and other neoplasms that are characterized by a rearrangement of the *TFE3* gene and the presence of the *ASPL*-*TFE3* fusion gene.
